# Model-specific tests on variance heterogeneity for detection of potentially interacting genetic loci

**DOI:** 10.1186/1471-2156-13-59

**Published:** 2012-07-18

**Authors:** Ludwig A Hothorn, Ondrej Libiger, Daniel Gerhard

**Affiliations:** 1 Institute of Biostatistics, Leibniz University Hannover, D–30419 Hannover, Germany; 2 Scripps Genomic Medicine, The Scripps Research Institute, La Jolla, CA–92037, USA

**Keywords:** Genetic association study, Quantitative traits, Interaction, Variance heterogeneity

## Abstract

**Background:**

Trait variances among genotype groups at a locus are expected to differ in the presence of an interaction between this locus and another locus or environment. A simple maximum test on variance heterogeneity can thus be used to identify potentially interacting single nucleotide polymorphisms (SNPs).

**Results:**

We propose a multiple contrast test for variance heterogeneity that compares the mean of Levene residuals for each genotype group with their average as an alternative to a global Levene test. We applied this test to a Bogalusa Heart Study dataset to screen for potentially interacting SNPs across the whole genome that influence a number of quantitative traits. A user-friendly implementation of this method is available in the R statistical software package multcomp.

**Conclusions:**

We show that the proposed multiple contrast test of model-specific variance heterogeneity can be used to test for potential interactions between SNPs and unknown alleles, loci or covariates and provide valuable additional information compared with traditional tests. Although the test is statistically valid for severely unbalanced designs, care is needed in interpreting the results at loci with low allele frequencies.

## Author’s summary

Interactions among alleles at variant sites in the genome or between alleles and the environment likely play an important role in determining complex traits such as blood pressure. However, sets of interacting loci are difficult to identify due to the large number of potential interactions that need to be tested. One approach that circumvents this difficulty is to identify loci that appear to take part in an interaction although their partners with which they interact are unknown. A SNP locus containing an allele that interacts with other alleles or the environment can be identified by the existence of a statistically significant difference in the variance of quantitative trait values among individuals who possess zero, one or two alleles at the locus. We describe an extension of Levene’s test, which was proposed to test variance heterogeneity. This new test has the advantage of providing information regarding the effect of specific alleles on variance heterogeneity, which can lead to formulating concrete, biologically relevant hypotheses about interacting alleles rather than just loci while controlling for type I error rate.

## Background

Statistical association between a biallelic marker and a quantitative trait is usually tested using either a two degree of freedom F-test in the one-way analysis of variance (ANOVA) [1], or a one degree of freedom F-test in a linear regression [2]. These approaches compare the means of quantitative trait values at genotype categories associated with a SNP locus (i.e., homozygous for major allele, heterozygous and homozygous for minor allele). While ANOVA is sensitive to any global heterogeneity, linear regression test is sensitive to the presence of an additive mode of inheritance. Less attention has been given to comparing the variances in the quantitative trait values associated with different genotype categories. Recently, [3] proposed using a standard Levene test [4] to identify variance heterogeneity due to potential interaction between a given locus and another allele at the same locus, alleles at different loci or the environment. They compared three global tests, namely the Bartlett-test, a rank modification of Bartlett test and Levene test particularly for non-normal distributed variables. Differences among the variances of quantitative trait values at each genotype category (denoted $\sigma_{j}^{2}$ with *j*=0,1,2 interacting alleles) may reflect an interaction [5]. In contrast to approaches that explicitly test specific gene-gene, e.g. by Bayesian partition methods [6] or gene-environment interactions, e.g. by multiple regression methods [7], methods that assess variance heterogeneity can be used to uncover loci that are not previously known to interact.

Levene test [8] tests a global null hypothesis $H_{0}\;\text{:}\;\sigma_{0}^{2}=\sigma_{1}^{2}=\sigma_{2}^{2}$ against the alternative that a difference exists between any pair of variances: $H_{1}\;\text{:}\;\exists j,j^{\prime \prime }\;\text{:}\;\;\sigma_{j}^{2}\neq \sigma_{j^{\prime \prime }}^{2},\;j\neq j^{\prime \prime }$. The test statistic consists of a quadratic form

(1) $$T_{\mathrm{Levene}}^{2}=\frac{(N-J)\overset{J}{\underset{j=0}{\sum }}n_{j}{\left(Z_{\mathrm{j.}}-\mathrm{Z..}\right)}^{2}}{(J-1)\overset{J}{\underset{j=0}{\sum }}{\left(Z_{\mathrm{ji}}-Z_{\mathrm{j.}}\right)}^{2}},\;N=\overset{J}{\underset{j=0}{\sum }}n_{j}$$

(with *J*=3) using the robust Levene residuals

(2) $$Z_{\mathrm{ji}}=\mathrm{abs}(Y_{\mathrm{ji}}-\mathrm{Median}(Y_{\mathrm{j.}})),$$

[8] with _*nj*_ quantitative trait observations _*Yij*_ per genotype *j*. The $T_{\mathrm{Levene}}^{2}$ is *F*-distributed with *d*_*f*1_=*J*and *d*_*f*2_=*N*− *J*.

This test is known to be relatively robust when data are not normally distributed. However, the main disadvantage of Levene’s test is that it can only be used to determine whether the group-specific variances differ among each other. In order to obtain a biologically or clinically relevant interpretation of the results, it is often valuable to additionally determine which pairs of genotype categories in particular exhibit statistically significant variance heterogeneity.

To this end, [3] considered using three two-sample *df*−1 tests for the three comparisons $\sigma_{0}^{2}$ vs. $\sigma_{12}^{2}$$\sigma_{1}^{2}$ vs. $\sigma_{02}^{2}$, and $\sigma_{2}^{2}$ vs. $\sigma_{01}^{2}$, where $\sigma_{jj^{\prime \prime }}^{2}$ denotes the variance estimator for the pooled groups *j*^*j″*^. However, these multiple tests do not control for the family-wise type I error rate *α*.

In this paper, we propose a Levene-type multiple contrast test, a novel approach comprised of a global test on variance heterogeneity as well as the three specific tests on pairwise variance heterogeneity using a maximum test of linear forms. We apply this test in a genome-wide fashion using a Bogalusa Heart Study dataset [9].

## Methods

### A Levene-type multiple contrast test

For the Levene-type transformed variable _*Zij*_and the factor *genotype* with the levels *j*=0,1,2 the following contrast test for the one-way layout is used:

(3) $$T_{k}^{\mathrm{Levene}}=\frac{\overset{J}{\underset{j=1}{\sum }}c_{\mathrm{kj}}{\bar{Z}}_{\mathrm{j.}}}{S\sqrt{\overset{J}{\underset{j=1}{\sum }}c_{\mathrm{kj}}^{2}/n_{j}}}$$

where *S* is the root of the common mean square error estimate of the linear model on the transformed data _*Zji*_ (absolute Levene residuals). Contrast coefficients _*ckj*_are used for the *k*=3 comparisons between the means of Levene residuals for each genotype with the average of the means. These comparisons coding for $\sigma_{0}^{2}$ vs. $\sigma_{12}^{2}$$\sigma_{1}^{2}$ vs. $\sigma_{02}^{2}$, and $\sigma_{2}^{2}$ vs. $\sigma_{01}^{2}$[10], are formulated in the 3×3 contrast matrix:

(4) $$(c_{\mathrm{kj}})=\left(\begin{array}{lll} -1 & 0.5 & 0.5\\ 0.5 & -1 & 0.5\\ 0.5 & 0.5 & -1 \end{array}\right).$$

A priori it is unknown which elementary test is mostly under the alternative. Therefore, the maximum of the test statistics $\max(T_{j})$ is used, implying a family-wise type-I-error rate *α*for all of the three comparisons.

Under the approximate assumption of multivariate normal distributed errors with a homogeneous global variance of the transformed variable _*Zij*_, the vector ^(_*Tj*_)*″*^ follows jointly a tri-variate *t*-distribution with *ν*=$\overset{J}{\underset{j=1}{\sum }}(n_{j}-1)$ degrees of freedom and a correlation matrix **R**=(_*ρk*^*k″*^_) given by its elements

(5) $$\rho_{kk^{\prime \prime }}=\frac{\overset{J}{\underset{j=1}{\sum }}c_{\mathrm{kj}}c_{k^{\prime \prime }j}/n_{j}}{\sqrt{\left(\overset{J}{\underset{j=1}{\sum }}c_{\mathrm{kj}}^{2}/n_{j}\right)\left(\overset{J}{\underset{j=1}{\sum }}c_{k^{\prime \prime }j}^{2}/n_{j}\right)}}.$$

Under the above assumptions the correlations _*ρk*^*k″*^_depend only on the contrast coefficients _*ckj*_and the sample sizes _*nj*_. This approach controls the familywise error rate *α*and reveals a reasonable power for unbalanced designs as long as the above assumptions hold true. It provides a global decision whenever any of the contrasts is under the alternative *and* additionally the elementary decisions by multiplicity-adjusted p-values for the three specific comparisons. Although simultaneous confidence intervals for both differences and ratios to the average are available as well [11], they will not be recommended since a genetic interpretation for the transformed variable _*Zji*_ is difficult. Recently, simultaneous confidence intervals for the pairwise ratios of variances were proposed using a maximum test on jackknifed $\log(s_{j}^{2})$[12]. This approach would provide an alternative when modified for arbitrarily unbalanced designs.

The question arises whether the quadratic form of the Levene test or the maximum contrast version of linear forms is more powerful. A general answer will not be available. For the comparison of normally distributed means, the least favorable configuration approach for $\mu_{j}-\bar{\mathrm{\mu .}}$ reveals higher power for the maximum of linear contrasts relative to the quadratic form of the F-test of the one-way analysis of variance [13]. Alternatively, a multiple contrast test based on pairwise comparisons of the genotype-specific variances *MC*^*TPairs*^ can be used by means of the following 3×3 contrast matrix:

(6) $$(c_{\mathrm{kj}})=\left(\begin{array}{lll} -1 & 1 & 0\\ 0 & -1 & 1\\ -1 & 0 & 1 \end{array}\right).$$

## Results and discussion

### Simulation Study

To validate the type-I-error control and compare the power between the multiple contrast test on the Levene scores and the Levene test, a small simulation study was conducted. Observations are sampled from a standard normal distribution for the three genotype groups. Overall sample size *N* is varied from 25 to 100, allocating the observations under assumption of Hardy-Weinberg equilibrium with allele frequencies *p* for the recessive allele of 0.5 and 0.75. To generate differences in variances, the variance is increased with the number of dominant alleles by a value of *δ* for an additive mode of inheritance (_*σ*0_=1,_*σ*1_=1 + *δ*,_*σ*2_=1 + 2*δ*). For a recessive and dominant model, the value of *δ*corresponding to the intermediate genotype is changed to 0 or 2 *δ*, respectively. For each parameter setting 10,000 simulation replications are performed. In Table 1 for the three approaches both control of type-I-error and comparable powers from a global test perspective can be concluded for the dominant mode of inheritance. However, the new *MC*^*TPairs*^and *MC*^*TAve*^approaches allows elementary decisions additionally. The related simulations for the additive and recessive modes are presented in the Appendix.

**Table 1:** Size and power comparison of the Levene test and two contrast alternatives given an dominant mode of inheritance

		**Levene**	***MC*^*TPairs*^**	***MC*^*TAve*^**						
**p**	**N**	***δ=* 0**	**0.5**	**1**	**0**	**0.5**	**1**	**0**	**0.5**	**1**
0.5	25	0.025	0.105	0.228	0.025	0.107	0.227	0.023	0.100	0.224
	50	0.036	0.398	0.792	0.036	0.392	0.790	0.035	0.403	0.794
	75	0.040	0.690	0.978	0.041	0.692	0.978	0.040	0.697	0.979
	100	0.039	0.868	0.999	0.038	0.869	0.999	0.039	0.871	0.999
0.75	25	0.015	0.126	0.268	0.015	0.131	0.282	0.013	0.118	0.263
	50	0.026	0.544	0.803	0.025	0.564	0.807	0.023	0.533	0.800
	75	0.036	0.842	0.954	0.035	0.850	0.955	0.032	0.831	0.954
	100	0.040	0.955	0.986	0.040	0.960	0.986	0.039	0.951	0.986

### Evaluation of a real data example

**Figure 1:**
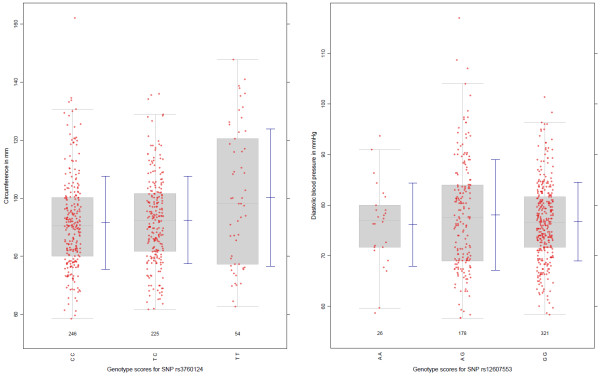
Boxplots including raw data for two selected SNPs and phenotypes of the Bogalusa Heart Study.

We evaluated the utility of the proposed test using data from Bogalusa Heart Study (BHS). The longitudinal study included genotype information on 525 unrelated individuals of European descent at 545,821 SNPs [12]. Twelve clinically-relevant quantitative traits were also measured for each study participant. We applied the multiple contrast test to each SNP throughout the genome. A number of SNP loci with a p-value smaller than <1^0−8^were identified for several quantitative traits whereas the number of SNPs with p-values below this threshold varied for different quantitative traits. For example, 23 SNP loci yielded p-values lower than that threshold when considering heart rate, while only 5 SNPs reached similar significance in the analysis involving body mass index. A thorough examination of the results showed that several significant tests occurred when the risk allele homozygote genotype group contained only a handful, i.e. 1,2,3, data points. Although the approach described in this paper is statistically valid in these situations, we selected only tests in which each genotype group contained at least one percent of all data points for further study. In the following text, we showcase two instances of variance heterogeneity in greater detail: rs3760124 and waist circumference, as well as rs12607553 and diastolic blood pressure; see the related box-plots in Figure 1.

The boxplots show relatively symmetric distributions of quantitative trait values in all genotype groups, thus ruling out the presence of outliers or extremely skewed distributions of trait values as sources of the observed variance heterogeneity. Furthermore, all three genotype categories contain a relatively large number of observed trait values resulting in reliable variance estimates.

**Figure 2:**
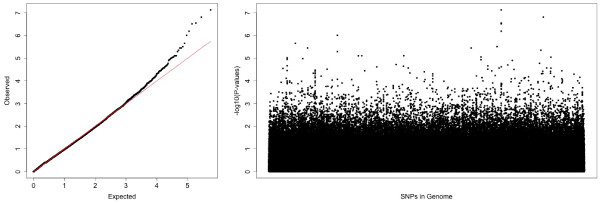
QQ- and Manhattan-plot for the phenotype waist circumference in the Bogalusa Heart Study.

It can be seen from Table 2 that our proposed test (labeled as *MC*^*TAve*^) yielded a result with greater statistical significance compared with the Levene global test. More importantly however, it allowed us to interpret the effect of the SNP alleles on variance heterogeneity. When we considered waist circumference, we found the variance heterogeneity to be most significant between the risk allele homozygous genotype group ($\sigma_{\mathrm{TT}}^{2}$), and the pooled variance for the two remaining genotype groups associated with the presence of at least one non-risk allele ($\sigma_{\mathrm{CC},\mathrm{CT}}^{2}$). In addition, we can see that the variance associated with the risk allele homozygotes is significantly higher in this example. Conversely, in the second example involving diastolic blood pressure, the variance in the heterozygotes $\sigma_{\mathrm{AG}}^{2}$ was significantly higher compared to homozygotes $\sigma_{\mathrm{AA},\mathrm{GG}}^{2}$. Such insights may prove to be valuable in forming hypotheses for further research, and can be obtained with the Levene-type multiple contrast test that we propose here. Furthermore, pairwise comparisons (labeled as *MC*^*TPairs*^) can also be performed (see Table 2). From the genome-wise analysis of the endpoint waist circumference the quantile-quantile plot and the Manhattan plot are presented in Figure 2 whereas SNPs with 5 or more individuals who are homozygous for the minor allele were considered. We see the expected inflationary behavior on a genome-wise level and the relatedness of the SNPs within the blocks. But only one SNP reveals is significant interaction on a global Bonferroni level.

**Table 2:** P values for original and multiple contrast Levene-type tests

**Trait**	**SNP**	**Test**	**Comparison**	**p-value**
waist circumference	rs3760124	Levene	global	3.4·1^0−07^
		*MC*^*TAve*^	$\sigma_{\mathrm{CC}}^{2}$ vs. $\sigma_{\mathrm{CT},\mathrm{TT}}^{2}$	2.7·1^0−01^
			$\sigma_{\mathrm{CT}}^{2}$ vs. $\sigma_{\mathrm{CC},\mathrm{TT}}^{2}$	1.5·1^0−01^
			$\sigma_{\mathrm{TT}}^{2}$ vs. $\sigma_{\mathrm{CC},\mathrm{CT}}^{2}$	*9.8*· *1*^*0*− *08*^
		*MC*^*TPairs*^	$\sigma_{\mathrm{CC}}^{2}$ vs. $\sigma_{\mathrm{CT}}^{2}$	9.6·1^0−01^
			$\sigma_{\mathrm{CC}}^{2}$ vs. $\sigma_{\mathrm{TT}}^{2}$	5.3·1^0−07^
			$\sigma_{\mathrm{CT}}^{2}$ vs. $\sigma_{\mathrm{TT}}^{2}$	4.4·1^0−07^
diastolic blood pressure	rs12607553	Levene	global	3.0·1^0−07^
		*MC*^*TAve*^	$\sigma_{\mathrm{AA}}^{2}$ vs. $\sigma_{\mathrm{AG},\mathrm{GG}}^{2}$	5.9·1^0−01^
			$\sigma_{\mathrm{AG}}^{2}$ vs. $\sigma_{\mathrm{AA},\mathrm{GG}}^{2}$	*1.2*· *1*^*0*− *07*^
			$\sigma_{\mathrm{GG}}^{2}$ vs. $\sigma_{\mathrm{AA},\mathrm{AG}}^{2}$	1.8·1^0−06^
		*MC*^*TPairs*^	$\sigma_{\mathrm{AA}}^{2}$ vs. $\sigma_{\mathrm{AG}}^{2}$	3.2·1^0−02^
			$\sigma_{\mathrm{AA}}^{2}$ vs. $\sigma_{\mathrm{GG}}^{2}$	9.9·1^0−01^
			$\sigma_{\mathrm{AG}}^{2}$ vs. $\sigma_{\mathrm{GG}}^{2}$	1.9·1^0−07^

An example R code for testing variance heterogeneity at a single SNP is provided in the Additional files 1 and 2. The multiplicity-adjusted p-values can be estimated by means of the R package multcomp [14]. Alternatively, a SAS procedure GLIMMIX can be used for a resampling based estimation of multiplicity-adjusted p-values [15].

## Conclusions

The important issue of missing heritability, which refers to the fact that common SNPs identified by genome-wide association studies as associated with a disease collectively explain only a small portion of the prevalence of this disease, may be due, in part, to the presence of unknown interactions among alleles at various SNP loci or environment, that affect the disease. The identification of such interactions is difficult, primarily because of the large number of potentially interacting pairs, trios, etc. of alleles and environmental variables that need to be tested. A feasible alternative, as suggested by [5], is to test individual loci for the evidence of their involvement in an interaction with other alleles, loci or covariates. The idea of assessing variance heterogeneity between three genotype groups at a particular SNP locus as evidence of a potential interaction is appealing for its simplicity. The Levene-type maximum contrast test proposed in this paper allows one to not only test for global variance heterogeneity, but also perform groupwise test that allows one to elucidate the effect of the individual alleles on quantitative trait variance. While this is an advantage over the standard Levene test, the price to pay is increased computing time. However, we were able to perform a genome-wide analysis of variance heterogeneity involving >500 individuals in a matter of minutes on a laptop computer. Parallelization can also be used to substantially decrease computation time requirements. R code implementing this test is available as part of the multcomp package.

Even the analysis of real data example illustrates the low specificity of the identified potential interactions. Care needs to be exercised in interpreting the results of this test in cases of low frequency variants or missing trait data, when one or more of the genotype groups contains an extremely small number of observed trait values. The issues surrounding the sensitivity and specificity of this approach in these potentially common cases is an area that needs further work.

## Appendix

The simulation results for the additive and recessive mode of inheritance are presented in Tables 3 and 4.

**Table 3:** Size and power comparison of the Levene test and two contrast alternatives given an additive mode of inheritance

		**Levene**	***MC*^*TPairs*^**	***MC*^*TAve*^**						
**p**	**N**	***δ=* 0**	**0.5**	**1**	**0**	**0.5**	**1**	**0**	**0.5**	**1**
0.5	25	0.026	0.102	0.225	0.026	0.099	0.209	0.025	0.102	0.221
	50	0.034	0.323	0.689	0.033	0.298	0.633	0.033	0.324	0.695
	75	0.037	0.554	0.926	0.038	0.511	0.897	0.037	0.561	0.928
	100	0.041	0.728	0.986	0.041	0.684	0.979	0.040	0.733	0.987
0.75	25	0.015	0.085	0.183	0.016	0.083	0.185	0.015	0.083	0.175
	50	0.029	0.283	0.637	0.028	0.286	0.641	0.026	0.265	0.613
	75	0.038	0.507	0.888	0.036	0.504	0.890	0.034	0.473	0.874
	100	0.041	0.678	0.976	0.041	0.682	0.977	0.041	0.650	0.972

**Table 4:** Size and power comparison of the Levene test and two contrast alternatives given an recessive mode of inheritance

		**Levene**	***MC*^*TPairs*^**	***MC*^*TAve*^**						
**p**	**N**	***δ=* 0**	**0.5**	**1**	**0**	**0.5**	**1**	**0**	**0.5**	**1**
0.5	25	0.022	0.212	0.496	0.022	0.217	0.518	0.020	0.206	0.479
	50	0.035	0.582	0.924	0.035	0.585	0.927	0.034	0.579	0.922
	75	0.037	0.809	0.989	0.038	0.811	0.990	0.040	0.805	0.989
	100	0.039	0.918	0.999	0.041	0.920	0.999	0.040	0.917	0.999
0.75	25	0.016	0.087	0.172	0.017	0.089	0.174	0.013	0.089	0.176
	50	0.032	0.206	0.430	0.030	0.207	0.433	0.027	0.217	0.443
	75	0.036	0.336	0.633	0.036	0.337	0.632	0.031	0.348	0.645
	100	0.039	0.444	0.772	0.037	0.444	0.774	0.037	0.458	0.782

## Authors’ contributions

LAH and DG derived the method. LAH and OL developed the software. OL performed the GWA analysis. LAH, OL and DG wrote the manuscript. All authors read and approved the final manuscript.

## Supplementary Material

Additional file 1 The R code. Click here for file

Additional file 2 **The data set**tg.rda**contains the subject-specific diastolic blood pressure data and the related genotype levels of the SNP rs12607553.** Click here for file
