# Supplementary material for: Model-specific tests on variance heterogeneity for detection of potentially interacting genetic loci
Source: BMC Genet. 2012 Jul 18;13:59. doi: 10.1186/1471-2156-13-59 (PMC3549778; doi:10.1186/1471-2156-13-59)
Supplement: Additional file 1 — The R code. [file 1471-2156-13-59-S1.pdf]

R-Code:

```
medrs12607553<-tapply(tg$dbp, tg$rs12607553, median) # group-specific medians
medrs12607553V<-medrs12607553[as.integer(tg$rs12607553)] # median for each subject
tg$tdbp<- abs(tg$dbp-medrs12607553V) # Levene transformation
library(multcomp)
lrs12607553<-lm(tdbp~rs12607553, data=tg) # fit one-way ANOVA linear model
summary(glht(lrs12607553, linfct=mcp(rs12607553 = "AVE"),
test=adjusted(maxpts=2500000, abseps=1e-10)))$test$pvalues # MCT-average
summary(glht(lrs12607553, linfct=mcp(rs12607553 = "Tukey"),
test=adjusted(maxpts=2500000, abseps=1e-10)))$test$pvalues # MCT pairs
```
